# Virtual Rehabilitation of the Paretic Hand and Arm in Persons With Stroke: Translation From Laboratory to Rehabilitation Centers and the Patient's Home

**DOI:** 10.3389/fneur.2021.623261

**Published:** 2021-01-28

**Authors:** Gerard Fluet, Qinyin Qiu, Jigna Patel, Ashley Mont, Amanda Cronce, Mathew Yarossi, Alma Merians, Sergei Adamovich

**Affiliations:** ^1^Rutgers Biomedical and Health Sciences, Newark, NJ, United States; ^2^New Jersey Institute of Technology, Newark, NJ, United States; ^3^Department of Physical Therapy, Movement and Rehabilitation Science, Northeastern University, Boston, MA, United States

**Keywords:** virtual reality, rehabilitation, stroke, hand, arm

## Abstract

The anatomical and physiological heterogeneity of strokes and persons with stroke, along with the complexity of normal upper extremity movement make the possibility that any single treatment approach will become the definitive solution for all persons with upper extremity hemiparesis due to stroke unlikely. This situation and the non-inferiority level outcomes identified by many studies of virtual rehabilitation are considered by some to indicate that it is time to consider other treatment modalities. Our group, among others, has endeavored to build on the initial positive outcomes in studies of virtual rehabilitation by identifying patient populations, treatment settings and training schedules that will best leverage virtual rehabilitation's strengths. We feel that data generated by our lab and others suggest that (1) persons with stroke may adapt to virtual rehabilitation of hand function differently based on their level of impairment and stage of recovery and (2) that less expensive, more accessible home based equipment seems to be an effective alternative to clinic based treatment that justifies continued optimism and study.

## Introduction

Virtual reality (VR) is an approach to human computer interface that utilizes multisensory feedback designed to foster a sense of immersion or agency in a simulated task or activity. Virtual environments, designed for the purposes of upper extremity rehabilitation in persons with stroke have been studied for more than 15 years. Unique aspects related to the control of sensory information make it an ideal method for presenting tasks in a manner consistent with the principles of use dependent neuroplasticity, affording scientists an opportunity to gain insight into the tenets of neuroplasticity and apply them to develop more effective rehabilitation interventions (1). Precise control of sensory presentations and task parameters as well as partial independence from the physics governing the real world make VR an efficient tool that is ideal for high volume practice, targeting motor skill development, in an enriched sensory environment. Animal and human studies have shown that the quantity, duration and intensity of training sessions are key variables in the design of interventions targeting structural changes at the synaptic level (2). Virtual rehabilitation is associated with substantially higher training volumes than traditional rehabilitation techniques in persons with stroke (3). The dynamic development of motor skills is a second requisite for adaptive changes in neural architecture (4). Virtual environments allow for an exquisite level of control over task parameters such as speed, accuracy demands and movement amplitude demands, which provide endless opportunities for incremental changes in task difficulty allowing therapists the ability to operantly shape progressively more normal motor skills (5).

Use of virtual environments to distort the relationship between actual participant movement and simulated movement can be leveraged in different ways to drive neuroplasticity in sensorimotor circuits at any impairment level. In severely impaired individuals with trace or absent hand movement, VR can be used to provide a modified form of mirror visual feedback training in which the unaffected limb is used to control a virtual avatar, visually representing movement of the affected limb. Mirror visual feedback training has been shown to enhance excitability in the ipsilateral (ipsilesional) hemisphere to moving hand via facilitation of compensoatory parieto-frontal networks (6–8). Virtual environments can also distort the relationship between actual participant movement and simulated movement. Trace movement of a body part can be scaled to produce meaningful avatar movement in a virtual environment that can accomplish meaningful tasks. For example, one or two degrees of finger flexion can move a virtual finger enough to strike a virtual piano key, producing a collision with the key that can be felt and movement of the key that can be seen in addition to the expected sound. This multimodal feedback of a scaled movement adds salience to small motor behaviors in profoundly impaired persons, and provides a reward signal for successful actions. Salience of sensory feedback about self-initiated actions is cited as a key requirement for neuroplasticity at any stage of recovery from stroke (9), and may play a crucial role early in the recovery period, when the levels of stroke-induced neuroplasticity are high (10), but the magnitude of upper extremity and particularly hand motor actions are often quite low (11). Furthermore, scaling the movement to provide a meaningful reward for sucessdful practice may help reinforce neural activity in motor and premotor areas of the practiced action. For less impaired individuals visuo-proprioceptive discordance can be created via hypometric or hypermetric feedback in order to promote sensorimotor learning. Sensorimotor motor learning using discordant feedback has been associated with increased excitability of the lesioned hemisphere that may induce a temporary enhancement of the neuroplastic effects of motor training (12, 13).

While the theoretical advantages of virtually simulated rehabilitation are many, the adoption of this technology in clinical settings has been slow. Initially, the cost of custom-made virtual rehabilitation systems was the most important initial barrier to adoption of this approach in clinical settings. This barrier hasbeen overcome by leveraging advancements made by the consumer electronics industries into lower cost rehabilitation technology. The other major hurdle that has been effectively overcome by technology advances is cyber-sickness which was experienced by early users of virtual environments. With these barriers addressed more domain specific limitations are being addressed. For example, the major technological limitation slowing development of the virtual rehabilitation of dexterity in persons with stroke (the focus of this paper) is the fidelity of lower cost motion capture systems (14).

## Virtual Rehabilitation of the Hemiparetic Upper Extremity

Rehabilitation of the hemiparetic hand caused by stroke has been one of the challenges that VR rehabilitation research has endeavored to overcome for a substantial portion of the field's existence. Early studies examined the ability of persons with chronic stroke to train safely and productively using this approach and comparisons between this approach, traditional interventions and repetitive task practice were conducted. Similar to the other labs in the growing field (5, 15), our group found that virtual rehabilitation interventions elicited clinically significant improvements in hand and arm motor function (16–21) that compared favorably to task–based interventions when measured using common clinical tests in persons with chronic stroke (22, 23). In addition, it was clear that our approach could modify specific aspects of motor function including finger fractionation, reaching trajectory length and smoothness, as well as arm stability during hand activity with virtually simulated motor training (18, 24). These changes have carried over to kinematic measures of transfer tasks utilizing real world objects (22) and improvements in activity level motor function (25) measured using standardized activity batteries such as the Wolf Motor Function Test and the Action Research Arm Test. Many labs including ours are working toward better measurement of participation level including 24 h activity monitoring and qualitative analysis of return to pre morbid roles in an attempt to overcome the varied success of identifying transfer to this level of function cited in the technology based rehabilitation literature (26, 27).

The early work in this area was followed by extensive work across the field. Large systematic reviews and meta-analyses support our assertion that virtual interventions elicit upper extremity function gains as measured by clinical test batteries that are comparable to, or better than traditionally presented interventions in persons with chronic stroke (26, 28). Demonstrating relative equivalence to in-person, physically presented rehabilitation is an important milestone for the field of virtual rehabilitation and a cause for heighteed focus in future studies. Many studies of virtual rehabilitation in persons with stroke are characterized by heterogenous subject pools and vaguely described interventions (29) which could lead to watered down effects and a poor understanding of the active ingredients of virtual interventions (30). Our group has endeavored to build on this initial success by attempting to identify patient populations, treatment settings and training schedules that will leverage virtual rehabilitation's unique strengths.

## Rehabilitation Early After Stroke

It is important to note that while consistent, measurable and statistically significant, the effect size of the gains demonstrated in studies of any rehabilitation intervention, virtual or otherwise were small and tended not to result in returns to full, pre cerebrovascular accident (CVA) levels of function or participation (31–33). The modest gains achieved by chronic stage upper extremity training and the identification of a critical period of heightened neuroplasticity (10) related to early recovery following a CVA, spurred many groups, including ours, to transition into the study of virtual rehabilitation during the acute and first few weeks of the early subacute stage (34) of recovery from stroke (11, 23, 35–37). Our group, successfully piloted an intervention in a small rehabilitation hospital. This study compared the outcomes of a group of subjects who recieved an in-patient rehabilitation program that started as few as five and as many as 15 days after stroke, with a second group receiving a similar rehabilitation program, supplemented with 8 h of VR based intervention, starting in the first few days after stroke, and a third group, that started the additional VR training between 30 and 90 days after their strokes (38). The safety and feasibility of this intensive training performed during an in-patient rehabilitation hospital stay was readily apparent. There were no adverse events associated with the training and no subjects missed regularly scheduled rehabilitation sessions due to their participation in our study. We found that the subjects performing additional VR based training of the hand in the early subacute phase after stroke demonstrated larger increases in motor performance when this change was normalized for overall recovery (average 6 month improvement in Normalized Box and Blocks Test score of 0.51 SD = 0.32) than subjects that only performed standard rehabilitation (0.43 SD = 0.32) or subjects that performed additional VR based training in the later subacute phase (0.13 SD = 0.10) (35). These findings differed from those summarized in the 2017 meta-analysis by Laver, but it is importatnt to note that Laver analyzed all studies in subjects <6 months post-stroke. This said, the two studies from this metanalysis that focused on the acute and first few weeks of the recovery stage both found non-significant trends favoring VR based interventions (23, 39). Our group has initiated a large scale clinicial trial addressing this topic as well as comparisons with a dose–matched program of traditional rehabilitation and a delayed onset program of virtual rehabilitation (40).

## Impact of VR Training on Cortical Excitatability

In an effort to gain insight into functional/electrophyisological changes made by the recovering brain and the impact of early, hand focused rehabilitation on these changes, we have employed transcranial magnetic stimulation (TMS) mapping in subsets of subjects participating in the pilot study we describe above. The first study compared 7 moderately impaired individuals who received an additional 8 h of VR/robotic intervention within 1 month post-stroke, to 6 similarily impaired individuals who did not receive additional hand focused rehabilitation (41). In both groups, there was an increase in ipsilesional first dorsal interosseous (FDI) map size from pre to post-training, and again from immediately post training to 1 month post-training suggesting that additional VR based hand rehabilitation might have had no impact on this aspect of the recovery process. This said, there was a stronger association between ipsilesional pre to 1 month FDI cortical map representation and long term (pre to 6 months post) improvements in Wolf Motor Function Test (WMFT) scores for the VR group (VR group r = −0.81, *p* = 0.049, UC group *r* = −0.31, *p* = 0.61). This may be due to the fact that the VR group, which received additional hand focused therapy in the very early recovery period, may have integrated expansion of the FDI motor map into better hand function. A companion study of 17 individuals who all received an additional 8 h hand focused VR/robotic training initiated within 3 months post lesion demonstrated a similar expansion of FDI area, and similar correlations between expansions in ipsilesional FDI map area and improvements in WMFT score (*r* = −0.75, *p* = 0.017) (42).

This correlation between lesioned hemisphere motor map expansion and hand function improvements following intensive hand training, but not usual care during the early recovery period, has been identified in studies by other labs (43, 44). Two groups using slightly different methods found no training related changes in map area (45, 46). These differing outcomes identified across our clinical, kinematic and neurophysiological studies examining the rehabilitation of persons with chronic stroke and those of our pilot studies of earlier virtual rehabilitation, have led us to initiate a larger study, adding a fourth group of subjects that perform an additional 10 h of traditional rehabilitation in an attempt to control for the timing of hand focused intervention, the dose of rehabilitation intervention, and the additive value of VR virtual reality-based rehabilitation (40).

## Identifying Persons Likely to Benefit From Virtual Rehabilitation

An additional issue related to the early rehabilitation of persons with CVA is the accurate identification of persons that might benefit from the additional hand focused rehabilitation. Rehabilitation prognoses for persons with stroke based on early motor mobility (ability to extend fingers, shoulder abduction) or presence of attention or neglect still fail to predict accurate motor recovery in a high percent of stroke survivors (47, 48). Rohafza et al. identified a multivariate model of four kinematic measures of movement collected in two virtual environments. This model predicted 56% of the variance (*p* = 0.042) in Jebsen Test of Hand Function change as the result of a 2 week training intervention (49). Rohafza et al. developed a similar multivariate model of measures of reach to grasp and object transport trajectory smoothness, hand opening, and trunk movement during a real object interaction test collected at baseline testing. This model predicted change scores in the 12 item Wolf Motor Function Test battery in a group of persons completing a 2 week virtual rehabilitation intervention (*r*^2^ = 0.74, *p* < 0.05) (50). The limited motor ability available to people earlier in the recovery process has led us to pursue other means of identifying patients that might benefit (42, 51). TMS-based measures of M1 excitability and electroencephalogram (EEG)-based cortical connectivity measures have shown to be promising biomarkers of recovery after stroke (52). Therefore, our current clinical trial aims to model recovery based on longitudinal measures of cortical excitability, cortical connectivity, and cortico-muscular connectivity (CMC) starting from the acute stage of stroke. Cortical excitability, connectivity, and CMC will be evaluated using measures of (1) motor evoked potential (MEP) – elicited by transcranial magnetic stimulation of the primary motor cortex and recording the response from target muscles, (2) cortical connectivity where EEG signals will be acquired during resting and active finger movement task, and (3) CMC where EEG and electromyographic (EMG) signals are acquired during active finger movement task. The only study we found that looked into changes in EEG activations from acute to chronic phases of stroke and their correlation with functional recovery was in ischemic rats (53). While MEP and cortical connectivity has been explored by other research groups as potential biomarkers of motor recovery (54), CMC during movement is a potential novel biomarker in a clinical setting, and has been explored by Kamp et al. as a marker of aging (55). Measures will be acquired: within 30 days post-stroke, before and after training,1 month post training and 4 and 6 months post-stroke. Data will be modeled to predict the extent of recovery and to understand if training early post stroke improves the prognosis of recovery.

## Rehabilitation of Persons With Severe Impairments

One adavantage afforded by virtual environments is the opportunity to manipulate sensory information (1). These manipuations can be utilized to enhance cortical excitability just prior to or during an activity (12) or to enhance the salience of training activities, maximizing long term neuroplasticity (2). A recent pilot study of ours tested a VR based intervention protocol for persons with severe hemiparesis leveraging some of these opportunities in an attempt to address the needs of this underserved population (56). There have been three studies examining virtual interventions in persons that are slightly less impaired during the chronic stage (Prange, Reinkensmeyer, Housman) Only the study by Housman suggests that virtual interventions might be more effective than traditional interventions. All three of these studies integrated robotic assistance into their interventions, but none utilized mirror priming activities. Our group's intervention included two priming activities. The first was a mirror activity designed to harness action observation networks. We attempted to strengthen the stimuli by allowing the subject to control the virtual image of their paretic hand by moving their non-paretic hand. This paired the image of their moving hand to a conscious intent to move. The second priming activity was movement based. This activity was designed to increase motor cortex excitability by moving the paretic hand passively with a cable actuated exoskeleton in an attempt to harness the impact of kinesthetic information on the lesioned motor cortex. Again we attempted to strengthen this stimuli by pairing it with a virtual image of the moving paretic hand, along with a haptically rendered collision with a ball at the end of the movement. An additional sensor-based pinching activity was also performed. This simulation allowed participants to utilize minimal active movement to perform a meaningful task, enhancing the salience of the intervention which optimized it as a stimuli for long term neuroplasticity. All but 1 of the nine subjects that enrolled in this study were able to control a cursor using a pinch grip measured with a sensitive force transducer. This active rehabilitation exercise was performed well before traditionally presented rehabilitation activities could be performed by these subjects. The group averaged a 30 point increase in Upper Extremity Fugl Meyer (UEFMA) score at 6 months (SD = 12). In addition, three of the seven subjects from this pilot demonstrated a >70% improvement in UEFMA score recovery at 6 months, exceeding the recovery predicted by prognostic algorithms (57, 58).

TMS mapping of a sub-set of patients from this more impaired group showed a different pattern of adaptation than the pattern identified in our less impaired subjects. In the more impaired subjects, extensive damage along the lesioned corticospinal tract made it impossible to elicit lesioned hemisphere motor evoked potentials at the impaired FDI, causing us to focus attention on the relationship between the contralesional motor cortex and the impaired UE. This sub-study showed an increase in the contralesional FDI map representation from pre to post training followed by a decrease from post to 1 month. The increase from pre to post intervention motor map area was associated with pre to 6 month increases in the UEFMA and maximum pinch force scores (56). We identified a similar pattern of increased cortical activation in the contralesional hemisphere during impaired finger movement in more impaired subjects, utilizing functional magnetic resonance imaging (fMRI) measures of cortical activation (51). These results are in line with several published articles highlighting that activity in the contralesional hemisphere is more pronounced in persons with larger lesions and more severe deficits - allowing for recovery in affected upper limb function via uncrossed corticospinal and reticulospinal tracts (59, 60) but diverge from another set of studies that do not demonstrate a relationship between contralesional changes and recovery in severely impaired subjects (61–63). It is important to note that this literature does not consider the impact of rehabilitation on this aspect of the recovery process. In a current clinical trial we are collecting cortical maps of both hemispheres at five points in the recovery process in persons with all levels of impairment (severe, moderate, and mild), that perform a standard rehab program as well as a standard rehab program plus added intensive hand training. We hope that this line of inquiry might help clarify the differing effects of rehabilitation across a wide variety of impairment levels, as this new study will include subjects with more extensive motor impairments than a majority of the major published upper extremity rehabilitation trials (40).

## Home Based Rehabilitation

We are currently focusing on incorporating our experience with haptics, virtual reality and gaming simulations to create a portable, self-manageable, home-based system, that allows patients to continue their hand and arm rehabilitation by integrating repetitive practice into their daily lives within their home environment. The camera-based system collects finger and arm position at a fidelity and speed that allows HoVRS to utilize real time algorithms to shape desired movement patterns. This high quality data also allows the system to provide kinematic data to therapists on or offline, allowing them to monitor and modify ‘patients’ rehabilitation programs remotely (64). Recent pilot study subjects demonstrated statistically and clinically significant improvements in hand motor performance as measured by clinical tests [mean UEMA improvement = 4.53 (SD = 2.3), Repeated Measures ANOVA (*p* < 0.001)] (65). They also made statistically significant error reductions during sine wave tracing tasks controlled by hand opening, wrist extension and forearm pronation measured by the home rehabilitation system (64). These outcomes are comparable with other studies of technology supported home based rehabilitation (66–70). Direct (albeit remote) supervision of subjects in these trials varied between very minimal (Standen) to extensive (Holden, Dakodian). We feel that clinically important gains demonstrated by our subjects are an important initial finding, when considering that this group of subjects did not incur transportation costs, required minimal supervision and used equipment that cost a small fraction of the cost of our lab-based system.

## Our Team; Ongoing Studies

Over the years our group has been a collaboration between biomedical engineers, physical therapists and neuroscientists, all sharing an interest in motor learning and neuroplasticity. Our approach is unique in the participation of all of these disciplines in the earliest stages of intervention technology design. Our main lab, which is housed in an engineering building, is equipped with lab grade kinematic measurement equipment, EEG and TMS equipment, several 3-D printers and a fittings shop. More recently, we have added satellite labs on the campuses of two rehabilitation hospitals. The balance of this paper will present unpublished and synthesized findings from previously published and unpublished studies that all examined virtual rehabilitation simulations in an attempt to present three contrasts: (1) adaptations made by persons with severe impairments Upper Extremity Fugyl Meyer Assessment (UEFMA) <20 and those with moderate to mild impairments (UEFMA 21–60) (2) long term adaptations made by subjects in the early subacute phase of recovery; and finally; (3) adaptations made by patients performing directly supervised interventions using costly facility based equipment with those of subjects using inexpensive equipment, in their homes, independently. Synthesis of these findings provide an overview of the progression of technological interventions for the hemiplegic hand and arm post stroke based on non-immersive virtual rehabilitation and, importantly, may provide a window into the most promising future pathways.

## Comparing Rehabilitation of Persons With Severe Impairments and Those With Milder Impairments

**Figure 1:**
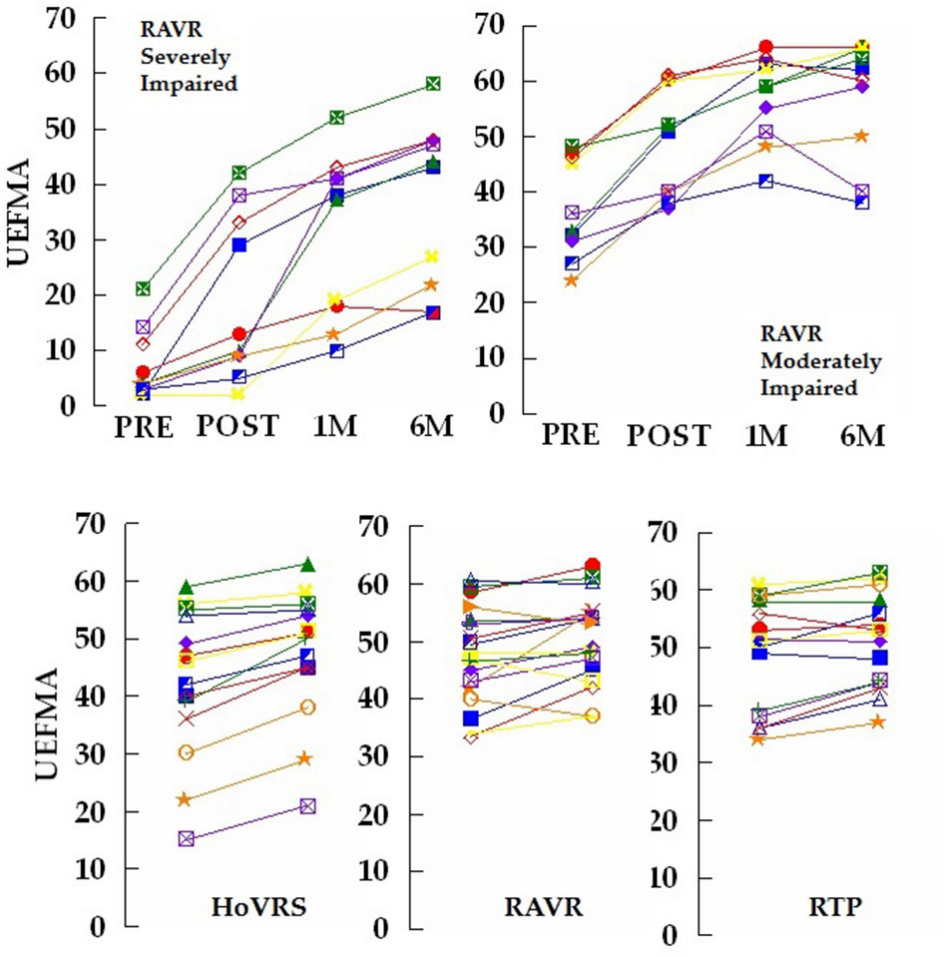
**Top left panel**: Total UEFMA score for 10 subjects that participated in a 2 week robot assisted virtual rehabilitation (RAVR) training protocol. Measurements taken pre – training and post-training as well as 1, 4, and 6 months post-stroke. These subjects all initiated training <30 days post-stroke and scored <20 on the UEFMA pre-training. Four of the subjects demonstrated an increase from pre to post-test, that exceeds the minimum clinically important difference (MCID) for persons with acute/early subacute stroke of 10 (71). Note the two distinct patterns of response to training and non-response to training at 1 month post-stroke. **Top right panel:** Total UEFMA score for 10 subjects that participated in the same RAVR training protocol. Measurements taken pre – training and post-training as well as 1, 4, and 6 months post-stroke. These subjects all initiated training <30 days post-stroke and scored more than 20 on the UEFMA pre-training. Seven of the subjects demonstrated an increase from pre to post-test, that exceeds the MCID. **Bottom Panel:** Total UEFMA score for 14 subjects that participated in a 12 week home virtual rehabilitation system (HoVRS) training protocol, 10 subjects that participated in a 2 week robot assisted virtual rehabilitation (RAVR) training protocol and 11 subjects that participated in a 2 week repetitive task practice (RTP) protocol. All subjects demonstrated residual impairments from stroke at least 6 months post-stroke and were tested immediately before and after training. Nine of the 14 HoVRS subjects demonstrated improvements that exceeded the MCID of 4.25 (72). Six of 17 RAVR subjects and five of 15 RTP subjects exceeded the MCID. Note the more homogenous improvements demonstrated by the subjects performing HoVRS training. RAVR and RTP subjects were described in detail in Ref. (22).

Our group has continued its examination of early rehabilitation in more impaired persons. Three additional subjects from our new study of early hand rehabilitation have completed the study through the 6 month data collection point and we have collected a total of 10 subjects with 6 month follow up that were less impaired as well. Figure 1 depicts the two recovery patterns for each group (unpublished data). Interestingly, the less impaired group shows a relatively consistent pattern of improvement with some ceiling effects and a bit of regression, while the more impaired group demonstrates two patterns. One subgroup makes improvements that exceed 40 points at follow up resulting in recoveries that exceeed overall recovery experienced by less impaired subjects. A second subgroup of more impaired subjects demonstrates more moderate improvement. It is important to note that the initial level of impairment in the more impaired group did not seem to determine which pattern the subjects would follow. Our initial impressions of these new data continue to suggest that some persons with fairly profound hand impairments may have rehabilitation potential that has not been leveraged in protocols examining early technology and traditionally presented UE rehabilitation. We argue that continued study of this population should be a major focus of the study of technology supported rehabilitation. Going forward we plan to explore the use of neuromodulatory techniques in this population. Most recently, our group has explored paired associative stimulation (PAS) which combines simultaneous central (via TMS) and peripheral (via neuromuscular electrical stimulation) stimulation on changes in cortical excitability during virtual mirror activities of the hand (73).

## Examining Rehabilitation During the Early Subacute Phases of Recovery

**Figure 2:**
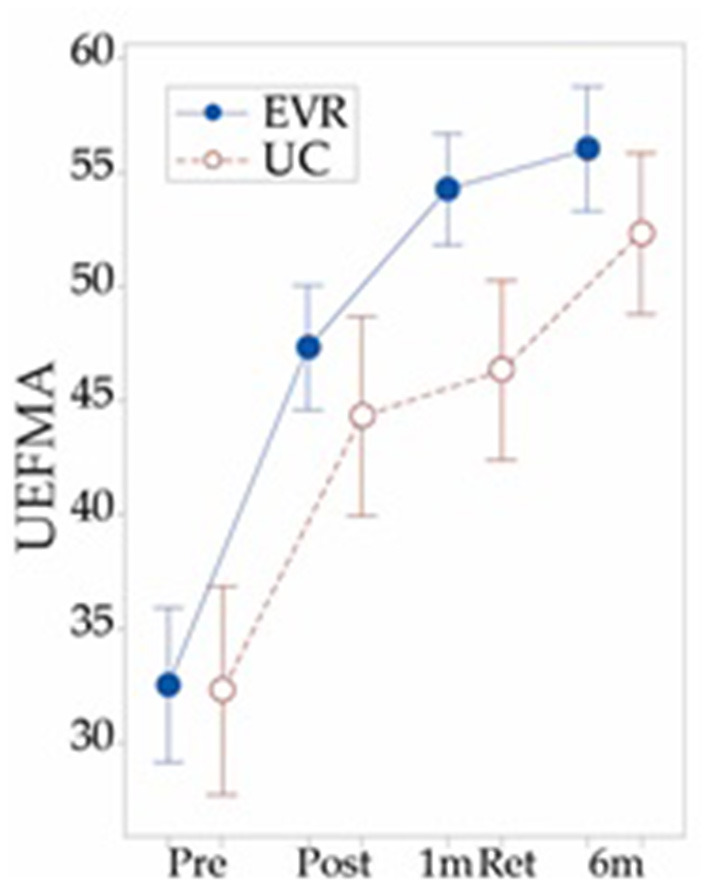
Total UEFMA score for 13 subjects that participated in a 10 session early virtual rehabilitation (EVR) training protocol added to their standard inpatient rehabilitation care, early home or outpatient rehabilitation and 7 subjects that performed usual care (UC) which consisted of standard inpatient rehabilitation care, early home or outpatient rehabilitation only. Both groups demonstrated changes that exceed the minimum detectable change of 5.25 from pre to post-test and from post-test to 6 month retention. Eight of the nine EVR subjects and eight of the 11 UC “subjects” improvements from pre to post-test exceeded the minimum clinically important difference of 10. Measurements taken pre – training and post-training (2–3 weeks after pre test for UC) as well as 1 month post-training and 6 months post-stroke. Note the differences in UEFMA score at 1 month post-training.

Longitudinal data collected from our pilot subjects in studies of hand-focused, early virtual rehabilitation after stroke suggest that the relative benefits of early intensive rehabilitation might be short - lived. Preliminary results indicate that 1 extra h of upper extremity training delivered by early virtual reality (EVR) (*n* = 10) during the 1 month post-stroke can be beneficial when compared to usual care (UC) (*n* = 11) at 1 month after the end of training, but this advantage seems to disappear at 6 months post-stroke (Unpublished data–See Figure 2). These findings align with several other studies of early rehabilitation (23, 36, 37) that do not observe longer term benefits in subjects who performed early rehabilitation of their hands. This said, the results in Figure 2 are more comparable to other studies of technology supported rehabilitation (74, 75) and a study of Constraint Induced Movement Therapy (20) that identified better outcomes than usual care during this early stage. Continued study of rehabilitation during this period is clearly warranted. Subjects in our ongoing trial of early rehabilitation are stratified by corticospinal tract integrity in an attempt to clarify this issue. We are also working toward evaluating an alternate hypothesis that additional high volume training might be necessary after patients are typically discharged from facility-based rehabilitation to preserve and further increase gains made during additional early training. Our group's home based system will offer an opportunity to study this continuation of the rehabilitation process.

## Comparing Home and Facility Based Rehabilitation

Our group's study of home based rehabilitation in persons with chronic stroke has demonstrated some interesting new trends. Adherence rates with these subjects has been comparable to other studies of technology supported, home based rehabilitation and better than traditional home based exercise programs in persons with stroke (64, 65). Interestingly, age and previous computer experience did not have an impact on adherence (76), similar to findings of a recent home based rehabilitation study (77). Additionally, our subjects have not demonstrated substantial decreases in compliance over the course of a 12 week intervention program. This diverges with the typical pattern of home exercise adherence which peaks 2 weeks into an intervention and decreases steadily after that point.

Comparing our current, home based virtual rehabilitation system (HoVRS) outcomes with our older work examining therapist supervised lab-based repetitive task practice (RTP) intervention and robot assisted virtual rehabilitation (RAVR), all in persons with chronic stroke offers some insight as well as some considerations for future study. In our 2015 paper comparing a robotically facilitated virtual rehabilitation intervention, to a therapist supervised circuit training intervention of 12 table-top repetitive task practice activities, both groups of subjects demonstrated a mean improvement of 2 points on the UEFMA. Both groups demonstrated inconsistent change patterns with some subjects improving, some staying the same and others regressing (22). This pattern differs from that of our home based subjects who demonstrated across the board improvement from pre to post-test with a mean improvement of more than 5 points (Unpublished data–See Figure 1).

The training schedules for these two interventions differed. The two lab based interventions were delivered in a 2 week period with 8, 3 h sessions. Our home based subjects averaged less therapy, closer to 18 total h, but the intervention occurred over 12 weeks. It is possible that a training schedule that distributes training time across a greater time period may be more conducive to motor improvements than a concentrated schedule with a larger volume of training. More rigorous testing will be required to determine if one approach to treatment or treatment schedule was definitively better. This said, the possibility that subjects using inexpensive equipment, in their homes, independently, might make similar to or better gains, than patients performing directly supervised interventions using costly equipment will have important implications for patient access to treatment and the cost of health care delivery. Additional further study will examine the feasibility of adding movement based priming to our home based interventions. Proof of concept testing of a low cost, admittance controlled finger training robot, that will utilize the same platform that presents our home based VR intervention is in progress (78).

## Conclusions

The anatomical and physiological heterogeneity of strokes and persons with stroke, along with the complexity of normal upper extremity movement make the possibility that any single treatment approach will become the definitive solution for all persons with upper extremity hemiparesis due to stroke unlikely. This situation and the non-inferiority level outcomes identified by many studies of virtual rehabilitation are considered by some to indicate that it is time to consider other treatment modalities. Data generated by our lab and others suggesting that (1) persons with stroke may adapt to virtual rehabilitation of hand function differently based on their level of impairment and stage of recovery and (2) that less expensive, more accessible home based equipment seems to be an effective alternative to clinic based treatment that justifies continued optimism and study.

## Data Availability Statement

The raw data supporting the conclusions of this article will be made available by the authors, without undue reservation.

## Ethics Statement

The studies involving human participants were reviewed and approved by Internal Review Board, Rutgers The State University of New Jersey. The patients/participants provided their written informed consent to participate in this study.

## Author Contributions

All authors listed have made a substantial, direct and intellectual contribution to the work, and approved it for publication.

## Conflict of Interest

GF, QQ, AMo, AC, AMe, and SA have applied for a patent for the Home Virtual Rehabilitation System. QQ, AMo, and AC have interests in NeuroTech3R, a company working toward bringing the Home Virtual Rehabilitation System to market. The remaining authors declare that the research was conducted in the absence of any commercial or financial relationships that could be construed as a potential conflict of interest.

## References

[1] iBadia BS Fluet GG Llorens R Deutsch JE. Virtual reality for sensorimotor rehabilitation post stroke: design principles and evidence. In: Reinkensmeyer D Dietz V editors. Neurorehabilitation Technology. Cham: Springer (2016). p. 573–603. 10.1007/978-3-319-28603-7_28

[2] Kleim AJ Jones AT. Principles of experience-dependent neural plasticity: implications for rehabilitation after brain damage. J Speech Lang Hear Res. (2008) 51:S225–39. 10.1044/1092-4388(2008/018) 18230848

[3] Rand D Givon N Weingarden H Nota A Zeilig G. Eliciting upper extremity purposeful movements using video games a comparison with traditional therapy for stroke rehabilitation. Neurorehabil Neural Repair. (2014) 28:733–9. 10.1177/1545968314521008 24515927

[4] Dimyan AM Cohen GL. Neuroplasticity in the context of motor rehabilitation after stroke. Nat Rev Neurol. (2011) 7:76–85. 10.1038/nrneurol.2010.200 21243015 PMC4886719

[5] Adamovich SV Fluet GG Tunik E Merians AS. Sensorimotor training in virtual reality: a review. NeuroRehabilitation. (2009) 25:29–44. 10.3233/NRE-2009-0497 19713617 PMC2819065

[6] Zhang JJ Fong KN Welage N Liu KP. The activation of the mirror neuron system during action observation and action execution with mirror visual feedback in stroke: a systematic review. Neural plasticity. (2018) 2018:2321045. 10.1155/2018/2321045 29853839 PMC5941778

[7] Manuweera T Yarossi M Adamovich S Tunik E. Parietal activation associated with target-directed right hand movement is lateralized by mirror feedback to the ipsilateral hemisphere. Front Hum Neurosci. (2019) 12:531. 10.3389/fnhum.2018.00531 30687047 PMC6333851

[8] Saleh S Adamovich SV Tunik E. Mirrored feedback in chronic stroke: recruitment and effective connectivity of ipsilesional sensorimotor networks. Neurorehabil Neural Repair. (2014) 28:344–54. 10.1177/1545968313513074 24370569 PMC3989389

[9] Tunney N Is there a best approach to the rehabilitation of adult hemiplegia? Phys Ther Rev. (2018) 23:348–54. 10.1080/10833196.2018.1539293

[10] Zeiler RS Krakauer WJ. The interaction between training and plasticity in the post-stroke brain. Curr Opin Neurol. (2013) 26:609. 10.1097/WCO.0000000000000025 24136129 PMC4012223

[11] Fluet GG Patel J Qiu Q Yarossi M Massood S Adamovich SV. Motor skill changes and neurophysiologic adaptation to recovery-oriented virtual rehabilitation of hand function in a person with subacute stroke: a case study. Disabil Rehabil. (2017) 39:1524–31. 10.1080/09638288.2016.1226421 27669997 PMC5368038

[12] Bagce HF Saleh S Adamovich SV Tunik E. Visuomotor gain distortion alters online motor performance and enhances primary motor cortex excitability in patients with stroke. Neuromodulation. (2012) 15:361–6. 10.1111/j.1525-1403.2012.00467.x 22672345 PMC3752791

[13] Tunik E Saleh S Adamovich SV. Visuomotor discordance during visually-guided hand movement in virtual reality modulates sensorimotor cortical activity in healthy and hemiparetic subjects. IEEE Trans Neural Syst Rehabil Eng. (2013) 21:198–207. 10.1109/TNSRE.2013.2238250 23314780 PMC3762883

[14] Guzsvinecz T Szucs V Lanyi SC. Suitability of the Kinect sensor and Leap Motion controller—a literature review. Sensors. (2019) 19:1072. 10.3390/s19051072 30832385 PMC6427122

[15] Sveistrup H. Motor rehabilitation using virtual reality. J Neuroeng Rehabilitation. (2004) 1:10. 10.1186/1743-0003-1-10 15679945 PMC546406

[16] Adamovich SV Fluet GG Merians AS Mathai A Qiu Q. Incorporating haptic effects into three-dimensional virtual environments to train the hemiparetic upper extremity. IEEE Trans Neural Syst Rehabilitation Eng. (2009) 17:512–20. 10.1109/TNSRE.2009.2028830 19666345 PMC2843820

[17] Adamovich SV Fluet GG Mathai A Qiu Q Lewis J Merians AS. Design of a complex virtual reality simulation to train finger motion for persons with hemiparesis: a proof of concept study. J Neuroeng Rehabilitation. (2009) 6:28. 10.1186/1743-0003-6-28 19615045 PMC2729310

[18] Merians AS Fluet GG Qiu Q Saleh S Lafond I Davidow A. Robotically facilitated virtual rehabilitation of arm transport integrated with finger movement in persons with hemiparesis. J Neuroeng Rehabilitation. (2011) 8:28. 10.1186/1743-0003-8-27 21575185 PMC3113321

[19] Fluet GG Merians AS Qiu Q Lafond I Saleh S Ruano V. Robots integrated with virtual reality simulations for customized motor training in a person with upper extremity hemiparesis: a case report. J Neurol Phys Ther. (2012) 36:28. 10.1097/NPT.0b013e3182566f3f 22592063 PMC4195597

[20] Adamovich SV Merians AS Boian R Lewis JA Tremaine M Burdea GS. A virtual reality—based exercise system for hand rehabilitation post-stroke. Presence. (2005) 14:161–74. 10.1162/1054746053966996 17271420

[21] Merians AS Poizner H Boian R Burdea G Adamovich S. Sensorimotor training in a virtual reality environment: does it improve functional recovery poststroke? Neurorehabil Neural Repair. (2006) 20:252–67. 10.1177/1545968306286914 16679503

[22] Fluet GG Merians AS Qiu Q Rohafaza M VanWingerden AM Adamovich S. Does training with traditionally presented and virtually simulated tasks elicit differing changes in object interaction kinematics in persons with upper extremity hemiparesis? Top Stroke Rehabil. (2015) 22:176–84. 10.1179/1074935714Z.0000000008 26084322 PMC4569092

[23] Silva Cameirão da M i Badia BS Duarte E Verschure PF Virtual reality based rehabilitation speeds up functional recovery of the upper extremities after stroke: a randomized controlled pilot study in the acute phase of stroke using the rehabilitation gaming system. Restor Neurol Neurosci. (2011) 29:287–98. 10.3233/RNN-2011-0599 21697589

[24] Fluet GG Merians AS Qiu Q Davidow A Adamovich SV. Comparing integrated training of the hand and arm with isolated training of the same effectors in persons with stroke using haptically rendered virtual environments, a randomized clinical trial. J Neuroeng Rehabilitation. (2014) 11:28. 10.1186/1743-0003-11-126 25148846 PMC4156644

[25] Cieza A Stucki G. The International Classification of Functioning Disability and Health: its development process and content validity. Eur J Phys Rehabil Med. (2008) 44:303–13. 18762740

[26] Laver KE Lange B George S Deutsch JE Saposnik G Crotty M. Virtual reality for stroke rehabilitation. Cochrane Database Syst Rev. (2017) 11:CD008349. 10.1002/14651858.CD008349.pub4 29156493 PMC6485957

[27] Mehrholz J. Is electromechanical and robot-assisted arm training effective for improving arm function in people who have had a stroke?: a cochrane review summary with commentary. Am J Phys Med Rehabilitation. (2019) 98:339–40. 10.1097/PHM.0000000000001133 30640290

[28] Lohse KR Hilderman CG Cheung KL Tatla S Loos Van H der M. Virtual reality therapy for adults post-stroke: a systematic review and meta-analysis exploring virtual environments and commercial games in therapy. PLoS ONE. (2014) 9:e93318. 10.1371/journal.pone.0093318 24681826 PMC3969329

[29] Maier M Ballester RB Duff A Oller DE Verschure PF. Effect of specific over nonspecific VR-based rehabilitation on Poststroke motor recovery: a systematic meta-analysis. Neurorehabil Neural Repair. (2019) 33:112–29. 10.1177/1545968318820169 30700224 PMC6376608

[30] Shrier I Platt RW Steele RJ. Mega-trials vs. meta-analysis: precision vs. heterogeneity? Contemp Clin Trials. (2007) 28:324–8. 10.1016/j.cct.2006.11.007 17188025

[31] Laver K George S Thomas S Deutsch JE Crotty M Virtual reality for stroke rehabilitation. Stroke. (2012) 43:e20–e21. 10.1161/STROKEAHA.111.642439 22713539

[32] Crosbie J Lennon S Basford J McDonough S. Virtual reality in stroke rehabilitation: still more virtual than real. Disabil Rehabilitation. (2007) 29:1139–46. 10.1080/09638280600960909 17613000

[33] Fluet GG Deutsch EJ. Virtual reality for sensorimotor rehabilitation post-stroke: the promise and current state of the field. Curr Phys Med Rehabilitation Rep. (2013) 1:9–20. 10.1007/s40141-013-0005-2 24579058 PMC3933268

[34] Bernhardt J Hayward KS Kwakkel G Ward NS Wolf SL Borschmann K. Agreed definitions and a shared vision for new standards in stroke recovery research: the stroke recovery and rehabilitation roundtable taskforce. Int J Stroke. (2017) 12:444–50. 10.1177/1747493017711816 28697708

[35] Fluet G Patel J Qiu Q Yarossi M Adamovich S Merians A Early versus delayed VR-based hand training in persons with acute stroke. In: Virtual Rehabilitation (ICVR), 2017 International Conference. Montreal (2017).p. 1–7. 10.1109/ICVR.2017.8007490

[36] Winstein CJ Wolf SL Dromerick AW Lane CJ Nelsen MA Lewthwaite R. Effect of a task-oriented rehabilitation program on upper extremity recovery following motor stroke: the ICARE randomized clinical trial. Jama. (2016) 315:571–81. 10.1001/jama.2016.0276 26864411 PMC4795962

[37] Dromerick A Lang C Birkenmeier R Wagner J Miller J Videen T. Very early constraint-induced movement during stroke rehabilitation (VECTORS): a single-center RCT. Neurology. (2009) 73:195–201. 10.1212/WNL.0b013e3181ab2b27 19458319 PMC2715572

[38] Merians A Yarossi M Patel J Qiu Q Fluet G Tunik E Examining VR/robotic hand retraining in an acute rehabilitation unit: a pilot study. In: Ibáñez J González-Vargas J María Azorín J Akay M Luis Pons J editors. Converging Clinical and Engineering Research on Neurorehabilitation II. Cham: Springer (2017). p. 437–41. 10.1007/978-3-319-46669-9_73

[39] Kong K-H. Efficacy of computer gaming in upper limb recovery after stroke: a randomized, controlled study. Cerebrovasc Dis. (2014) 36:18. 10.1159/000367674 27098818

[40] Merians A Fluet G Qiu Q Yarossi M Patel J Mont A. Hand focused upper extremity rehabilitation in the subacute phase post-stroke using interactive virtual environments. Front Neurol. 11:573642. 10.3389/fneur.2020.573642 33324323 PMC7726202

[41] Patel J Fluet G Qiu Q Yarossi M Merians A Tunik E. Intensive virtual reality and robotic based upper limb training compared to usual care, associated cortical reorganization, in the acute and early sub-acute periods post-stroke: a feasibility study. J Neuroeng Rehabilitation. (2019) 16:28. 10.1186/s12984-019-0563-3 31315612 PMC6637633

[42] Yarossi M Patel J Qiu Q Massood S Fluet G Merians A. The association between reorganization of bilateral m1 topography and function in response to early intensive hand focused upper limb rehabilitation following stroke is dependent on ipsilesional corticospinal tract integrity. Front Neurol. (2019) 10:28. 10.3389/fneur.2019.00258 30972004 PMC6443957

[43] Boake C Noser EA Ro T Baraniuk S Gaber M Johnson R. Constraint-induced movement therapy during early stroke rehabilitation. Neurorehabil Neural Repair. (2007) 21:14–24. 10.1177/1545968306291858 17172550

[44] Ro T Noser E Boake C Johnson R Gaber M Speroni A. Functional reorganization and recovery after constraint-induced movement therapy in subacute stroke. Neurocase. (2006) 12:50–60. 10.1080/13554790500493415 16517515

[45] Platz T Kaick Van S Löller M Freund S Winter T Kim IH. Impairment–oriented training and adaptive motor cortex reorganisation after stroke: a fTMS study. J Neurol. (2005) 252:1363–71. 10.1007/s00415-005-0868-y 15965585

[46] Sánchez G-J Amengual JL Rojo N de las Heras VM Montero J Rubio F. Plasticity in the sensorimotor cortex induced by Music-supported therapy in stroke patients: a TMS study. Front Hum Neurosci. (2013) 7:28. 10.3389/fnhum.2013.00494 24027507 PMC3759754

[47] Rehme AK Volz LJ Feis DL Eickhoff SB Fink GR Grefkes C. Individual prediction of chronic motor outcome in the acute post-stroke stage: behavioral parameters versus functional imaging. Human Brain Mapp. (2015) 36:4553–65. 10.1002/hbm.22936 26381168 PMC4619153

[48] Stinear CM Byblow WD Ackerley SJ Smith MC Borges VM Barber PA. PREP2: a biomarker-based algorithm for predicting upper limb function after stroke. Ann Clin Trans Neurol. (2017) 4:811–20. 10.1002/acn3.488 29159193 PMC5682112

[49] Rohafza M Fluet GG Qiu Q Adamovich S. Correlation of reaching and grasping kinematics and clinical measures of upper extremity function in persons with stroke related hemiplegia. In: Engineering in Medicine and Biology Society (EMBC), 2014 36th Annual International Conference of the IEEE. Chicago, IL (2014). p. 3610–13. 10.1109/EMBC.2014.6944404 PMC5638303 25570772

[50] Rohafza M Fluet GG Qiu Q Adamovich S. Correlations between statistical models of robotically collected kinematics and clinical measures of upper extremity function. Conf Proc IEEE Eng Med Biol Soc. (2012) 2012:4120–3. 10.1109/EMBC.2012.6346873 23366834 PMC4552313

[51] Saleh S Fluet G Qiu Q Merians A Adamovich SV Tunik E. Neural patterns of reorganization after intensive robot-assisted virtual reality therapy and repetitive task practice in patients with chronic stroke. Front Neurol. (2017) 8:28. 10.3389/fneur.2017.00452 28928708 PMC5591400

[52] Boyd LA Hayward KS Ward NS Stinear CM Rosso C Fisher RJ. Biomarkers of stroke recovery: consensus-based core recommendations from the stroke recovery and rehabilitation roundtable. Int J Stroke. (2017) 12:480–93. 10.1177/1747493017714176 28697711 PMC6791523

[53] Zhang S-j Ke Z Li L Yip S-p Tong K-y. EEG patterns from acute to chronic stroke phases in focal cerebral ischemic rats: correlations with functional recovery. Phys Meas. (2013) 34:28. 10.1088/0967-3334/34/4/423 23524534

[54] Rosso C Lamy J-C Prediction of motor recovery after stroke: being pragmatic or innovative? Curr Opin Neurol. (2020) 33:482–7. 10.1097/WCO.0000000000000843 32657889

[55] Kamp D Krause V Butz M Schnitzler A Pollok B. Changes of cortico-muscular coherence: an early marker of healthy aging? Age. (2013) 35:49–58. 10.1007/s11357-011-9329-y 22037920 PMC3543740

[56] Patel J Qiu Q Yarossi M Merians A Massood S Tunik E. Exploring the impact of visual and movement based priming on a motor intervention in the acute phase post-stroke in persons with severe hemiparesis of the upper extremity. Disabil Rehabilitation. (2017) 39:1515–23. 10.1080/09638288.2016.1226419 27636200 PMC5355001

[57] Stinear CM Byblow WD Ackerley SJ Smith M-C Borges VM Barber PA Proportional motor recovery after stroke. Stroke. (2017) 48:795–8. 10.1161/STROKEAHA.116.016020 28143920

[58] Krakauer WJ Marshall SR. The proportional recovery rule for stroke revisited. Ann Neurol. (2015) 78:845–7. 10.1002/ana.24537 26435166

[59] Chieffo R Inuggi A Straffi L Coppi E Rosa J-G Spagnolo F. Mapping early changes of cortical motor output after subcortical stroke: a transcranial magnetic stimulation study. Brain Stimulation. (2013) 6:322–9. 10.1016/j.brs.2012.06.003 22776700

[60] Veldema J Kösl B Nowak DA. Motor recovery of the affected hand in subacute stroke correlates with changes of contralesional cortical hand motor representation. Neural Plasticity. (2017) 2017:6171903. 10.1155/2017/6171903 28286677 PMC5329670

[61] Ferreri F Guerra A Rossini PM. Neurophysiological markers of plastic brain reorganization following central and peripheral lesions. Arch italiennes de biologie. (2014) 152:216–38. 10.12871/00039829201443 25987182

[62] Traversa R Cicinelli P Bassi A Rossini PM Bernardi G. Mapping of motor cortical reorganization after stroke. A brain stimulation study with focal magnetic pulses. Stroke. (1997) 28:110–7. 10.1161/01.STR.28.1.110 8996498

[63] Freundlieb N Philipp S Drabik A Gerloff C Forkert ND Hummel FC. Ipsilesional motor area size correlates with functional recovery after stroke: a 6-month follow-up longitudinal TMS motor mapping study. Restor Neurol Neurosci. (2015) 33:221–31. 10.3233/RNN-140454 25503508

[64] Qiu Q Cronce A Patel J Fluet GG Mont A Merians AS. Development of the Home based Virtual Rehabilitation System (HoVRS) to remotely deliver an intense and customized upper extremity training. J Neuroeng Rehabilitation. (2020) 7:155. 10.21203/rs.3.rs-64042/v1 33228709 PMC7685660

[65] Fluet GG Qiu Q Patel J Cronce A Merians AS Adamovich SV. Autonomous use of the home virtual rehabilitation system: a feasibility and pilot study. Games Health J. (2019) 8:432–8. 10.1089/g4h.2019.0012 31769724 PMC7133442

[66] Standen PJ Threapleton K Connell L Richardson A Brown DJ Battersby S. Patients' use of a home-based virtual reality system to provide rehabilitation of the upper limb following stroke. Phys Ther. (2015) 95:28. 10.2522/ptj.20130564 25212521

[67] Popović DM Kostić DM Rodić ZS Konstantinovi LM. Feedback-mediated upper extremities exercise: increasing patient motivation in poststroke rehabilitation. BioMed Res Int. (2014) 2014:520374. 10.1155/2014/520374 24991557 PMC4060770

[68] Chang E Zhao X Cramer SC. Home-based hand rehabilitation after chronic stroke: randomized, controlled single-blind trial comparing the MusicGlove with a conventional exercise program. J Rehabil Res Dev. (2016) 53:28. 10.1682/JRRD.2015.04.0057 27532880

[69] Holden MK Dyar TA Cimadoro L-D. Telerehabilitation using a virtual environment improves upper extremity function in patients with stroke. IEEE Trans Neural Syst Rehabilitation Eng. (2007) 15:36–42. 10.1109/TNSRE.2007.891388 17436874

[70] Dodakian L McKenzie AL Le V See J Fuhrhop K-P Burke E Quinlan. A home-based telerehabilitation program for patients with stroke. Neurorehabil Neural Repair. (2017) 31:923–33. 10.1177/1545968317733818 29072556 PMC5734923

[71] Shelton FD Volpe BT Reding M. Motor impairment as a predictor of functional recovery and guide to rehabilitation treatment after stroke. Neurorehabil Neural Repair. (2001) 15:229–37. 10.1177/154596830101500311 11944745

[72] Page SJ Fulk GD Boyne P. Clinically important differences for the upper-extremity Fugl-Meyer Scale in people with minimal to moderate impairment due to chronic stroke. Phys Ther. (2012) 92:791–8. 10.2522/ptj.20110009 22282773

[73] Alokaily AO Yarossi M Fluet GG Tunik E Adamovich SV. The effect of movement phase on the contralaterally coordinated paired associative stimulation-induced excitability. Conf Proc IEEE Eng Med Biol Soc. (2018) 2018:3080–3. 10.1109/EMBC.2018.8512931 30441045 PMC6457650

[74] Saposnik G Teasell R Mamdani M Hall J McIlroy W Cheung D. Effectiveness of virtual reality using Wii gaming technology in stroke rehabilitation: a pilot randomized clinical trial and proof of principle. Stroke. (2010) 41:1477–84. 10.1161/STROKEAHA.110.584979 20508185 PMC4879973

[75] Piron L Tombolini P Turolla A Zucconi C Agostini M Dam M. Reinforced feedback in virtual environment facilitates the arm motor recovery in patients after a recent stroke. Virtual Rehabilitation. (2007) 2007:121–3. 10.1109/ICVR.2007.436215115455905

[76] Fluet G Qiu Q Cronce A Sia E Blessing K Wohn D Participant adherence to a video game based tele-rehabilitation program – a mixed-methods case series. In: Press Hayre C Muller D Shere M editors. Virtual Reality in Health and Rehabilitation. Boca Raton, FL: CRC Press (2020). p. 169–184. 10.1201/9780429351365-13

[77] Cramer SC Dodakian L Le V See J Augsburger R McKenzie A. Efficacy of home-based telerehabilitation vs in-clinic therapy for adults after stroke: a randomized clinical trial. JAMA Neurol. (2019) 76:1079–87. 10.1001/jamaneurol.2019.1604 31233135 PMC6593624

[78] Zhou X Mont A Adamovich S Evaluation of a 1-DOF hand exoskeleton for neuromuscular rehabilitation. In: International Symposium on Computer Methods in Biomechanics and biomedical engineering. New York City, NY (2019). p. 384–97. 10.1007/978-3-030-43195-2_32

