# Supplementary material for: Virtual Rehabilitation of the Paretic Hand and Arm in Persons With Stroke: Translation From Laboratory to Rehabilitation Centers and the Patient's Home
Source: Front Neurol. 2021 Jan 28;12:623261. doi: 10.3389/fneur.2021.623261 (PMC7876436; doi:10.3389/fneur.2021.623261)
Supplement: Supplementary file 1 [file Data_Sheet_1.PDF]

Sample sizes

Comparing rehabilitation of persons with severe impairments and those with milder impairments

Subjects:

Inclusion:

Persons 5 – 30 days post hemorrhagic or ischemic stroke

Age: 30–90 years old,

Cognition: Able to follow instructions

Severity: Upper Extremity Fugyl Meyer Assessment Score ≥0/66 and ≤49/66

Intact cutaneous sensation

Exclusion:

Too ill to tolerate training

Severe spatial neglect

Severe proprioceptive impairment

Modified Ashworth Score ≥ 3; elbow, wrist or finger flexors

Intervention:

Both groups received standardized inpatient rehabilitation therapy plus 10h of intensive therapy focusing on the hand using robotically facilitated rehabilitation interventions presented in virtual environments and initiated 5–30 days post-stroke.

All subjects utilized five to six simulations chosen from a library of sixteen simulations designed for persons with stroke. All subjects performed at least two hand simulations and at least two shoulder and elbow simulations. Subjects without active hand movement performed contralaterally controlled simulations designed to increase cortical excitability of the lesioned hemisphere. Subjects with active, but moderate to severely impaired motor function performed robotically facilitated simulations and movement amplitude scaled simulations that allowed them to perform simulations at an approximately 80% success rate. Subjects were weaned from robotic assistance and difficulty was increased as performance improved. Subjects with more mild impairments performed simulations without assistance. Difficulty was scaled based on speed, accuracy demands and motor control demands of the tasks (eg. Gross hand opening and closing progressed to finger individuation; single joint movements progressed to complex multi-joint movements).

Comparison:

Subjects are grouped based on their pre test UEFMA score. The severe impairment group subjects scored 0 to 20 on the UEFMA and the moderate group scored more than twenty on the UEFMA.

Outcomes:

Measurements were taken pre – training and post training as well as one, four and six months post stroke.

Examining rehabilitation during the early subacute phases of recovery

Subjects:

Inclusion:

Persons 5 – 30 days post hemorrhagic or ischemic stroke

Age: 30–90 years old,

Cognition: Able to follow instructions

Severity: Upper Extremity Fugyl Meyer Assessment Score ≥10/66 and ≤49/66

Exclusion:

Medical instability

Motor impairment from prior stroke

Severe spatial neglect

≥1 on the NIHSS limb ataxia item

Severe proprioceptive impairment

Severe spasticity

Previous history of orthopedic or neurological deficits limiting upper extremity function

Intervention:

Both groups received standardized inpatient rehabilitation therapy. The Usual Care Group The Early Robotic / VR Therapy (EVR) performed an extra 10h of intensive therapy focusing on the hand using robotically facilitated rehabilitation interventions presented in virtual environments which were initiated 5–30 days post-stroke. All subjects utilized five to six simulations chosen from a library of sixteen simulations designed for persons with stroke. All subjects performed at least two hand simulations and at least two shoulder and elbow simulations. Subjects without active hand movement performed contralaterally controlled simulations designed to increase cortical excitability of the lesioned hemisphere. Subjects with active, but moderate to severely impaired motor function performed robotically facilitated simulations and movement amplitude scaled simulations that allowed them to perform simulations at an approximately 80% success rate. Subjects were weaned from robotic assistance and difficulty was increased as performance improved. Subjects with more mild impairments performed simulations without assistance. Difficulty was scaled based on speed, accuracy demands and motor control demands of the tasks (eg. Gross hand opening and closing progressed to finger individuation; single joint movements progressed to complex multi-joint movements).

Comparison:

Subjects were randomly assigned to the EVR or UC groups prior to pretesting.

Outcomes:

Measurements were taken pre – training and post training as well as one and six months post stroke by assessors that are blinded to group assignment.

Comparing home and facility based rehabilitation

Subjects:

Inclusion:

Persons at least 6 months post hemorrhagic or ischemic stroke

Age: 18 to 80 years old

Severity:

Robot assisted virtual rehabilitation (RAVR) and repetitive task practice (RTP) groups: - at least 20° of active wrist extension and at least 10° of active finger extension.

Home virtual rehabilitation system (HOVRS) group: UEFMA score ≥ 15.

Exclusion:

Aphasia

Significant hemi-sensory inattention

Intervention:

RAVR:

Subjects were trained for 3 hours per day, four consecutive days per week, for 2 weeks in a lab setting. Subjects performed three shoulder and elbow simulations and 3 wrist and hand simulations. The simulations integrated virtual environments and haptic robotics using a system specifically designed for stroke rehabilitation. Three activities simulated functional activities and three were games. Activity difficulty was modified by the system using a patient performance algorithm or was adjusted manually using a success based criteria.

RTP:

Subjects were trained for 3 hours per day, four consecutive days per week, for 2 weeks in a lab setting. Subjects performed twelve different functional activities, each for fifteen minutes. Activities were designed to train movement patterns similar to those trained by the RAVR intervention. Each activity was trained at eight to twelve difficulty levels that were scaled based on object size (larger objects are often easier to for fine motor activities and vice versa for gross motor activities), task complexity, object weight, movement speed and movement amplitude. When subjects completed a task, they proceeded to a higher difficulty version of the same task, until the activity rotation time was complete. Subsequent

sessions started at one or two difficulty levels below the most difficulty level attempted in the previous session.

HoVRS:

Subjects trained in their homes for three months. They did not have set schedules, but agreed at screening to train at least sixty minutes per week. The group averaged thirty hours of training over the three month session. Subjects were taught to use the system in their homes. There were in home follow up sessions to introduce new games and perform modifications to games that could not be performed remotely. Subjects had the ability to communicate with study personel via phone, text or internet seven days per week. A majority of technical support and follow-ups were conducted via the internet. Subjects played three to six games, at least one for the shoulder, one for the wrist and one for the shoulder and elbow. Activity difficulty was modified by the system using a patient performance algorithm or presented in a series of levels that gradually increased in difficulty and complexity.

Comparison:

Subjects for the RTP group were recruited consecutively. Subjects from the RAVR arm of the study were chosen from a single arm of a larger study of subjects participating in a study of virtually simulated UE rehabilitation. HoVRS subjects were recruited consecutively.

Outcome Measurement:

Measurements were taken pre – training and post training.
